# Isolation of canine *Anaplasma phagocytophilum* strains from clinical blood samples using the *Ixodes ricinus* cell line IRE/CTVM20

**DOI:** 10.1016/j.vetmic.2012.10.021

**Published:** 2013-03-23

**Authors:** Viktor Dyachenko, Christine Geiger, Nikola Pantchev, Monir Majzoub, Lesley Bell-Sakyi, Inke Krupka, Reinhard K. Straubinger

**Affiliations:** a Institute for Infectious Diseases and Zoonoses, Department for Veterinary Sciences, Faculty for Veterinary Medicine, LMU Munich, Veterinaerstr. 13, 80539 Munich, Germany; b IDEXX Vet Med Lab, Moerikestr. 28/3, 71636 Ludwigsburg, Germany; c Institute of Veterinary Pathology, Center for Clinical Veterinary Medicine, Faculty for Veterinary Medicine, LMU Munich, Veterinaerstr. 13, 80539 Munich, Germany; d The Roslin Institute and Royal (Dick) School of Veterinary Studies, University of Edinburgh, Easter Bush, Midlothian EH25 9RG, UK

**Keywords:** Tick cell lines, *Anaplasma phagocytophilum*, IRE/CTVM20, Dog, Electron microscopy

## Abstract

*Anaplasma phagocytophilum* is an intracellular tick-borne rickettsial pathogen, which causes granulocytic anaplasmosis in various species of livestock and companion animals and also in humans. Previously *A. phagocytophilum* has been isolated and propagated in cell lines derived from the tick *Ixodes scapularis* and in the human promyelocytic cell line HL60. In this study we used the *Ixodes ricinus*-derived cell line IRE/CTVM20 to isolate and propagate two new canine strains of *A. phagocytophilum*.

Blood samples were collected by veterinarians from two dogs, one from Germany and the other from Austria. Suspicion of clinical canine granulocytic anaplasmosis was raised by the treating veterinarians and after confirmation of *A. phagocytophilum* infection by real-time PCR, buffy coat cells were isolated and co-cultivated with IRE/CTVM20 cells maintained at 28 °C in L15/L15B medium.

In the tick cells, rickettsial inclusions were first recognised after 86 days of incubation. Electron microscopic examination of tick cells infected with one of the isolates revealed cytoplasmic vacuoles containing pleomorphic organisms with individual bacteria enveloped by a bilayer membrane. Sequencing of 16S rRNA genes confirmed the isolation of *A. phagocytophilum* and showed the highest identity to the *A. phagocytophilum* human HZ strain. The two *A. phagocytophilum* isolates were passaged several times in IRE/CTVM20 cells and transferred to the *I. scapularis* cell line ISE6. This confirms for the first time the successful establishment and continuous cultivation of this pathogen in *I. ricinus* cells as well as infectivity of these canine strains for *I. scapularis* cells.

## 1 Introduction

*Anaplasma phagocytophilum* is an intracellular rickettsial pathogen, which belongs to the alpha-proteobacteria. *A. phagocytophilum* includes the pathogens previously known as *Ehrlichia phagocytophila* in ruminants, *Ehrlichia equi* in equines and human granulocytic ehrlichiosis (HGE) agent in humans (Rikihisa, 2011), which were reclassified based on molecular genetic analysis (Dumler et al., 2001). However, variable pathogenicity for different mammalian hosts as well as genetic variation have been observed in *A. phagocytophilum*, suggesting a complex ecology of this pathogen (Pusterla et al., 1999, 2001; Foley et al., 2009; Woldehiwet, 2010; Scharf et al., 2011). Possibly this pathogen comprises a variety of distinct ecotypes that have evolved a range of strategies to enable their survival (Bown et al., 2009).

The bacterium is transmitted by ixodid ticks (mainly *I. ricinus* in Europe and *I. scapularis* or *I. pacificus* in the USA) and can cause a disease with nonspecific, sometimes severe, clinical signs known as granulocytic anaplasmosis in horses (Engvall et al., 1996), dogs (Egenvall et al., 1997), cats (Bjoersdorff et al., 1999) and humans (Dumler et al., 2005), and as tick-borne fever in ruminants (Woldehiwet, 2010). It was shown in experimentally infected animals that persistent infection occurs with recurrent periods of bacteraemia lasting up to 2 months in dogs (Scorpio et al., 2011), up to 4 months in horses (Franzen et al., 2009) and up to 12 months in sheep (Thomas et al., 2012).

*A. phagocytophilum* is a challenging intracellular pathogen, requiring an appropriate host cell for its propagation, as no axenic cultures have yet been reported. In mammalian hosts *A. phagocytophilum* is found mainly in granulocytes, but it can also infect bone marrow progenitor and endothelial cells (Rikihisa, 2011). The establishment of continuous tick cell lines has facilitated the propagation and isolation of new strains of organisms such as *Anaplasma* and *Ehrlichia* as reviewed earlier (Bell-Sakyi et al., 2007). The first successful attempts to isolate *A. phagocytophilum* of human and equine origin were performed using the *I. scapularis* cell line IDE8 and the human promyelocytic cell line HL-60 (Goodman et al., 1996; Munderloh et al., 1996b). The *I. scapularis* cell lines IDE8 and ISE6 have been widely used to isolate and propagate *A. phagocytophilum* from blood of different mammalian species as well as from tick tissues (Munderloh et al., 1999; Woldehiwet et al., 2002; Massung et al., 2006; Zweygarth et al., 2006; Silaghi et al., 2011). The use of tick cell lines for the isolation of different *A. phagocytophilum* strains seems to be independent of the ecotype, as the ruminant-specific *Ap*-variant 1 has been isolated into *I. scapularis* cell lines, whereas isolation attempts in HL-60 cells were not successful (Massung et al., 2006). Little is known about the suitability of the *I. ricinus* cell lines IRE/CTVM20 and IRE/CTVM19 for isolation and growth of *A. phagocytophilum.* It was shown that propagation in the IRE/CTVM19 cell line is possible (Pedra et al., 2010); however, there are no reports of use of *I. ricinus* cell lines for isolation of *A. phagocytophilum* strains. Here we describe for the first time the successful isolation of two new strains of *A. phagocytophilum* (*Ap*Muc01c and *Ap*Muc02c) from canine blood samples using the *I. ricinus* cell line IRE/CTVM20.

## 2 Materials and methods

### 2.1 Blood samples and preparation of infected white blood cells (WBC)

Blood samples were collected by veterinarians from two dogs, one from Germany and the other from Austria. A suspicion of clinical canine granulocytic anaplasmosis was raised by the treating veterinarians and blood samples were submitted to a private veterinary laboratory (IDEXX Vet Med Lab) for comprehensive examination. The dog from Austria (2-year old, female) had a history of previous tick infestation. At the time of presentation, this dog showed fever (40.5 °C), lethargy, recumbency, abnormal behaviour and vomiting. The abnormal laboratory findings were thrombocytopenia, leukopenia, lymphopenia, hypoalbuminaemia (with decreased total protein) and the blood showed a low specific *A*. *phagocytophilum* antibody titre (IgG) of 1:100 in *A. phagocytophilum*-immunofluorescence antibody assay (IFA) performed as described previously (Dyachenko et al., 2012). The animal recovered quickly after oral doxycycline treatment. The dog from Germany (4-year old, castrated, male) showed no clinical signs at the time of presentation, but a marked thrombocytopenia (70 G/l; reference range of 150–500 G/l) was observed on a preoperative screening examination. Further specific tests for *A. phagocytophilum* were initiated thereafter (IFA for antibodies (IgG) was negative; titre <1:50) and, after doxycycline treatment, thrombocyte levels were within the reference range (at approx 5 weeks after initial examination). No inclusions suspected of being *A. phagocytophilum* morulae were detected microscopically on a routine examination of Giemsa-stained blood smears from either dog, but the presence of *A. phagocytophilum* DNA in the blood samples was confirmed by real-time PCR (Ct-values of 17 for the Austrian dog and 22 for the dog from Germany; no Ct-values were obtained in negative controls and/or healthy animals).

White blood cells were harvested from the blood samples at the Institute for Infectious Diseases and Zoonoses one week after collection, using the following protocol: approximately 500 μl of each blood sample was loaded onto Ficoll 1077 and centrifuged at 700 × *g* for 30 min at 4 °C to separate erythrocytes. The top layer containing WBC was subjected to hypotonic shock with 10 ml prechilled 0.2% NaCl for 30 s to lyse remaining erythrocytes. The osmolarity was restored by adding an equal volume of 1.6% NaCl. The WBC suspension was centrifuged at 250 × *g* for 6 min and the cells were washed once in Hanks’ balanced salt solution without calcium and magnesium.

### 2.2 Tick cell cultures

The *I. ricinus* embryo-derived cell line IRE/CTVM20 (Bell-Sakyi et al., 2007) was maintained in a 1:1 mixture of L-15 (Leibovitz) medium and L-15B medium (Munderloh and Kurtti, 1989) supplemented with 12% foetal calf serum (FCS), 10% tryptose phosphate broth (TPB, Sigma–Aldrich), 0.05% bovine lipoprotein (MP Biomedicals) and 2 mM l-glutamine (PAA) (L-15/L-15B). The cells were cultured in sealed containers in ambient air at 28 °C. The isolated WBC were co-cultivated with IRE/CTVM20 cells in 25 cm^2^ cell culture flasks at 28 °C using 5 ml of the same medium as above with the addition of 10 mM HEPES and 0.1% NaHCO_3_. The medium subsequently used for infected IRE/CTVM20 cells was buffered to pH 7.5 with 1 N NaOH. After 4 weeks of culturing infected IRE/CTVM20 cells, the concentration of FCS was reduced to 5%. The *I. scapularis* embryo-derived cell line ISE6 (Kurtti et al., 1996) was maintained at 32 °C in L-15B300 medium (Munderloh et al., 1999) supplemented with 5% FCS, 10% TPB, 0.1% bovine lipoprotein and 2 mM l-glutamine. For culturing, *A. phagocytophilum*-infected ISE6 cells were grown in 25 cm^2^ cell culture flasks in ambient air at 34 °C and the medium was additionally supplemented with 10 mM HEPES and 0.1% NaHCO_3_, and buffered to pH 7.5 with 1 N NaOH. Medium was changed once a week for both cell lines. Giemsa-stained cytocentrifuge smears, prepared as follows, were examined fortnightly by light microscopy. Infected IRE/CTVM20 cell cultures were gently resuspended, an aliquot of cell suspension was diluted 1:5 with appropriate cell culture medium and 200 μl aliquots of diluted suspension were used to prepare the cytocentrifuge smears. For infected ISE6 cell cultures, 200–800 μl aliquots of undiluted supernatant (depending on turbidity) were used.

### 2.3 Real-time-PCR

To monitor the growth of *A. phagocytophilum* in the cultures, real-time-PCR was performed. DNA was extracted (Qiagen blood & tissue kit) from 200 μl of cultured cell suspension and analysed by real-time PCR as previously described (Courtney et al., 2004). Results were evaluated by comparison of Ct values.

### 2.4 Polymerase chain reaction and sequencing

Total DNA was extracted from IRE/CTVM20 cell suspensions using the QIAamp DNA Blood Mini kit (Qiagen, Germany) according to the manufacturer's instructions. A 1400-bp fragment of the 16S rRNA gene was amplified using primers, proof reading polymerase and reaction conditions as previously described (Zhou et al., 2010). Amplicons were purified and submitted for sequencing to Eurofins MWG Operon (Ebersberg, Germany). Each PCR product was sequenced three times in both directions using PCR primers. Finally, 1348-bp long partial 16S ribosomal RNA sequences were deposited in GenBank™ under accession numbers JX173651 and JX173652 from *Ap*Muc01c and *Ap*Muc02c, respectively.

### 2.5 Transmission electron microscopy

*A. phagocytophilum*-infected IRE/CTVM20 cells (*Ap*Muc01c from original culture and first subculture) and control uninfected IRE/CTVM20 cells were gently resuspended, 1.5 ml aliquots of cell suspension were transferred to microcentrifuge tubes and centrifuged at 200 × *g* for 5 min. The cell pellets were resuspended in 2.5% glutaraldehyde solution in Sorenson's sodium phosphate buffer and fixed for 1 h at 4 °C. Following fixation the cells were washed three times in Sorenson's sodium phosphate buffer, postfixed in 1% osmium tetroxide for 1 h at 4 °C and then washed three times in Sorenson's sodium phosphate buffer, dehydrated in an ascending acetone series and embedded in epoxy resin. Ultrathin sections (70–80 nm) were stained with uranyl citrate and lead citrate and examined with an EM10 transmission electron microscope (Zeiss, Oberkochen, Germany).

## 3 Results

**Fig. 1:**
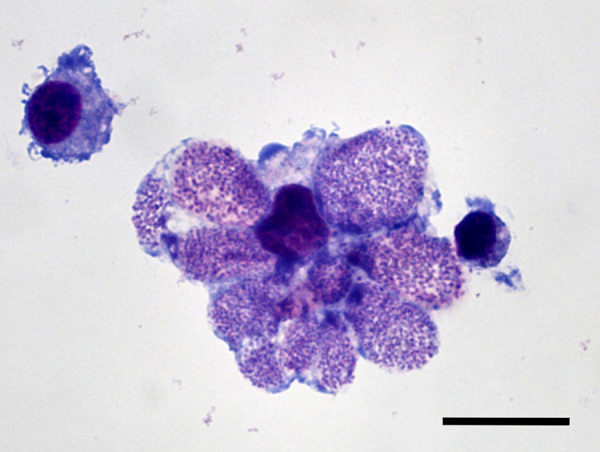
*A. phagocytophilum* (strain *Ap*Muc01c)-infected IRE/CTVM20 cell culture showing one heavily infected tick cell containing several endosomes with individual bacteria (centre), and two uninfected cells. Scale bar represents 10 μm.

Rickettsial inclusions in the tick cells were first recognised after 86 days of incubation for both *A. phagocytophilum* strains, *Ap*Muc01c and *Ap*Muc02c, isolated from canine blood samples from Germany and Austria respectively. The infected cells contained one or more large vacuoles filled with numerous bacteria (Fig. 1). The examination of DNA extracted from infected cell suspensions by quantitative real-time PCR confirmed multiplication of *A. phagocytophilum* by decrease of Ct values over time. Once established, the *A. phagocytophilum* strains were subsequently subcultured every 4–6 weeks. For this purpose, 500 μl aliquots of cell suspension containing nearly all infected cells were transferred into new 25 cm^2^ flasks containing uninfected IRE/CTVM20 cells in 5 ml cell culture medium (final dilution 1:10). The *A. phagocytophilum Ap*Muc01c and *Ap*Muc02c strains were passaged five and three times respectively in IRE/CTVM20 cells.

The *I. scapularis* cell line ISE6 was also inoculated with cell suspensions from IRE/CTVM20 cultures infected with both *A. phagocytophilum* strains. The infection resulted in rapid development of inclusions containing *Anaplasma* organisms in the ISE6 cells and finally in lysis of the cell monolayer. Subsequently both strains were passaged three times in ISE6 cells. When either strain was subcultured into new IRE/CTVM20 and ISE6 cells simultaneously and with equal inoculum size, the development of bacterial inclusions was observed firstly in ISE6 cells and then in IRE/CTVM20 cells about one week later. No differences were observed between the two strains in microscopical appearance and multiplication rate.

Sequencing of the 16S rRNA gene of both strains confirmed the isolation of *A. phagocytophilum*, as the sequences showed the highest identity to the *A. phagocytophilum* entries available in GenBank. The 16S rRNA sequences isolated from the two strains were however not identical to each other as one single nucleotide transition from G to A at position 376 was observed in *Ap*Muc01c, corresponding to the sequenced 16 rRNA gene of the human *A. phagocytophilum* strain HZ (GenBank accession number CP000235.1:1057470–1058902).

**Fig. 2:**
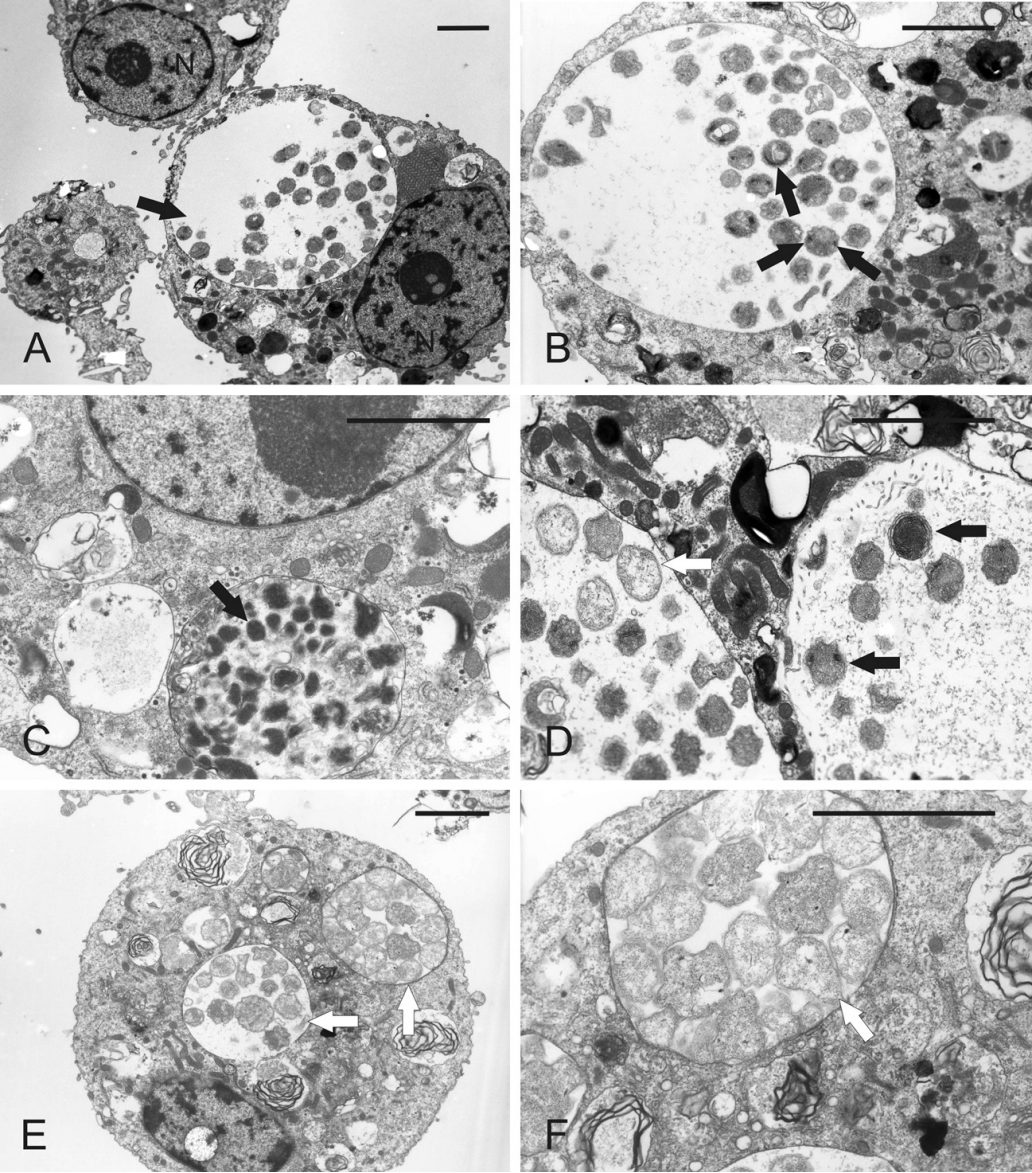
Ultrastructure of *A. phagocytophilum* (strain *Ap*Muc01c) in IRE/CTVM20 cells examined by transmission electron microscopy. (A) Vacuole (black arrow) containing single bacteria (N: nucleus). (B) *A. phagocytophilum* inclusion in a tick cell showing electron-dense forms; note the double polar dots possibly representing a condensed nucleoside (black arrows). (C) Highly electron-dense organisms (black arrow) in a vacuole. (D) Electron-dense (black arrows) and reticulate forms (white arrow), which were visible in two vacuoles of an infected cell; some individual bacteria are surrounded by a double membrane. (E) and (F) Vacuoles containing electron-lucent reticulate organisms (white arrows); note the electron-dense pinpoint structures in some bacteria. Scale bars in all pictures represent 2 μm.

Electron microscopy was performed on the original culture of *Ap*Muc01c in IRE/CTVM20 cells and on its first subculture. Examination of the infected tick cells revealed cytoplasmic vacuoles of different sizes containing mainly coccoid and pleomorphic organisms with ruffled outer membranes (Fig. 2). Overall the organisms ranged in size from approximately 0.44 μm to 1.60 μm. In most of the inclusions bacteria appeared electron-dense and their size ranged from 0.44 μm to 0.82 μm (median 0.62 μm, *n* = 30), shown in Fig. 2A and B. However, some of those bacteria appeared highly electron dense (Fig. 2C). Nearly all bacteria were enveloped by a distinct double membrane; rarely one or more additional membranes were seen surrounding the bacteria (Fig. 2D). An intriguing observation was the appearance of electron-dense material inside the coccoid organisms, sometimes as bipolar points or thin crescent-shaped structures (Fig. 2B). Two out of ten infected IRE/CTVM20 cells examined contained some smaller vacuoles with larger (size ranged from 0.5 μm to 1.6 μm, median 0.75 μm, *n* = 16) pleomorphic reticulate organisms (Fig. 2E and F). These bacteria were easily distinguishable from the electron-dense forms, and sometimes contained small electron-dense pinpoint structures. In some cases these bacteria were arranged close to each other in a single vacuole, while sometimes the reticulate forms were present in vacuoles together with electron dense forms. No intracellular bacteria were seen in preparations of uninfected IRE/CTVM20 cultures.

## 4 Discussion

Disease caused by *A. phagocytophilum* in ruminants in Europe has been recognised for over 80 years, and early experiments demonstrated that *I. ricinus* was the vector (Woldehiwet, 2006). However, a European isolate of *A. phagocytophilum* was only cultivated continuously *in vitro* for the first time, in the *I. scapularis* cell line IDE8, a decade ago (Woldehiwet et al., 2002). This delay, compared to the progress made with New World *A. phagocytophilum* isolates in cells from the North American vector *I. scapularis*, could be explained by the limited availability of cell lines derived from the European vector *I. ricinus*. Several *I. ricinus* embryo-derived cell lines are now available (Simser et al., 2002; Bell-Sakyi, 2004; Bell-Sakyi et al., 2007); in the present study one of these lines, IRE/CTVM20, was used as an alternative to ISE6 and IDE8 cells to successfully isolate and propagate two European isolates of *A. phagocytophilum* from canine blood samples.

The *I. scapularis* cell lines IDE8 and ISE6 have been shown to be a universal medium for isolating and cultivating *A. phagocytophilum*, applicable independent of the mammalian host species or the pathogen ecotype. This is shown by the successful isolation of strains of equine, canine, ruminant, human and tick origin using *I. scapularis* cell lines (Munderloh et al., 1996b, 1999; Woldehiwet et al., 2002; Massung et al., 2006; Silaghi et al., 2011). The ability of *A. phagocytophilum* organisms originating from a variety of host species to grow in *I. scapularis* as well as in *I. ricinus* cell lines confirms that the pathogen utilises the same mechanisms to invade and propagate in cells of both tick species (Pedra et al., 2010). The *I. scapularis*-derived IDE8 and ISE6 cell lines seem to be highly permissive to many Alphaproteobacteria (*e.g. Anaplasma marginale*, *Ehrlichia ruminantium*, *Rickettsia felis*), which normally utilise vectors other than *Ixodes* spp. ticks (Bell-Sakyi, 2004; Munderloh et al., 1996a; Sunyakumthorn et al., 2008). *E. ruminantium* and *A. marginale* have been grown in the *I. ricinus* cell line IRE/CTVM18 (Bell-Sakyi, 2004; Zweygarth et al., 2006) and *Rickettsia monacensis* has been propagated in the *I. ricinus* cell line IRE11 (Simser et al., 2002). It remains to be ascertained how suitable the *I. ricinus*-derived cell lines IRE/CTVM20 and IRE/CTVM19 are for propagation of pathogens not normally transmitted by *Ixodes* spp.

This is the first report describing the successful isolation of *A. phagocytophilum* from blood samples using cell culture derived from an indigenous European vector species: *I. ricinus*. To isolate the pathogen from canine blood samples the buffy coat cells were co-cultivated with IRE/CTVM20 cells at 28 °C. Infected tick cell cultures were maintained for 86 days until the intracellular inclusions were visible. This long incubation period is unfavourable compared to routine isolation procedures based on ISE6 and IDE8 cell lines. The latter are maintained at 34 °C and *Anaplasma* inclusions can become visible as early as 7 or 11 days after inoculation (Woldehiwet et al., 2002; Munderloh et al., 1996b). However, it should be considered that the incubation period that is needed for successful isolation depends on the bacterial load in the original sample material. In the present case, *A. phagocytophilum* morulae were not observed microscopically in Giemsa-stained blood smears, although the blood samples were positive in real-time PCR with Ct values of 22 and 17 for the isolated strains *Ap*Muc01c and *Ap*Muc02c, respectively. Depending on bacterial loads, the Ct values in *msp2* real time PCR of blood from *A. phagocytophilum* infected dogs can be as low as Ct 13, suggesting a high bacteraemia, or as high as Ct 35 in lightly infected dogs (unpublished observations). Furthermore, blood samples were first processed for *Anaplasma* isolation one week after collection from the dogs, by which time many of the bacteria in the sample would no longer be viable. In addition, growth of the established *A. phagocytophilum* strains (first passage) in IRE/CTVM20 occurred more slowly at 28 °C than in ISE6 at 34 °C. Both circumstances (low bacterial load and lower growth rate at 28 °C) resulted finally in prolonged incubation times before *Anaplasma* inclusions could be detected microscopically in IRE/CTVM20 cells. On the other hand lower incubation temperatures may overcome the problem with bacterial contaminations to some extent, particularly if no antibiotics are used. The problem with bacterial contaminations is known and was described elsewhere (Silaghi et al., 2011).

Sequencing of the 16S rRNA and quantitative real-time PCR confirmed the isolation and propagation of *A. phagocytophilum* in IRE/CTVM20 cells. The sequences of both isolated strains were highly similar to each other but not identical due to one transition from A to G. A sequencing artefact cannot be excluded in this case, but seems to be unlikely as the PCR was performed with proof reading polymerase and sequencing reactions were performed 3 times each in both directions. The resultant chromatograms presented clear sequencing reactions without any double peaks or sequencing artefacts. Both *A. phagocytophilum* strains were isolated from dogs with either signs of acute granulocytic anaplasmosis including fever, lethargy, recumbency, lymphopaenia, hypoalbuminaemia and thrombocytopenia (Austrian dog) or abnormal clinicopathological findings such as marked thrombocytopenia (German dog). It is not clear if the two strains could have different biological features. Due to the high conservation of the 16S rRNA no biological characteristics can be inferred, so both strains should be further characterised by sequencing of other genetic markers such as *msp4* and *ankA* (de la Fuente et al., 2005; Scharf et al., 2011).

Electron microscopical examination of thin sections of infected cells revealed bacteria of different morphological types which can be summarised into three groups: large electron-lucent highly irregularly shaped organisms, small electron-dense coccoid organisms and small highly electron-dense coccoid organisms. The electron-lucent and electron-dense forms of *A. phagocytophilum* are known as reticulate cells and dense-cored cells respectively, and represent different forms of biphasic development (Munderloh et al., 1999; Troese and Carlyon, 2009). In general the ultrastructural appearance of canine *Ap*Muc01c strain in IRE/CTVM20 cells was similar to that of *Anaplasma* sp. from white-tailed deer, equine *A. phagocytophilum* and *Ap*-Variant1 strains of *A. phagocytophilum* as well as *A. marginale* in the *I. scapularis*-derived IDE8 and ISE6 cell lines (Munderloh et al., 1996b, 2003; Blouin and Kocan, 1998; Massung et al., 2007). The larger electron-lucent organisms corresponded to reticulate cells, which are considered to be proliferating forms and were reported in *A. phagocytophilum* and *Anaplasma* sp. from white-tailed deer cultured in ISE6 cells (Munderloh et al., 1999, 2003). However, most of the inclusions contained electron-dense organisms in large vacuoles, which are believed to be infective forms. Many of these organisms contained electron-dense material appearing as two polar dots, which could represent condensed nucleoside.

We conclude that the *I. ricinus* cell line IRE/CTVM20 can be added to the list of tick cell lines suitable for isolation and continuous cultivation of *A. phagocytophilum* derived from clinical samples. The procedure for *A. phagocytophilum* strain isolation using IRE/CTVM 20 is particularly beneficial in cases where no antibiotics can be used.

## Conflict of interest

The authors declare that they have no conflict of interest.

## References

[bib0005] Bell-Sakyi L. (2004). *Ehrlichia ruminantium* grows in cell lines from four ixodid tick genera. J. Comp. Pathol..

[bib0010] Bell-Sakyi L., Zweygarth E., Blouin E.F., Gould E.A., Jongejan F. (2007). Tick cell lines: tools for tick and tick-borne disease research. Trends Parasitol..

[bib0015] Bjoersdorff A., Svendenius L., Owens J.H., Massung R.F. (1999). Feline granulocytic ehrlichiosis – a report of a new clinical entity and characterisation of the infectious agent. J. Small Anim. Pract..

[bib0020] Blouin E.F., Kocan K.M. (1998). Morphology and development of *Anaplasma marginale* (Rickettsiales: Anaplasmataceae) in cultured *Ixodes scapularis* (Acari: Ixodidae) cells. J. Med. Entomol..

[bib0025] Bown K.J., Lambin X., Ogden N.H., Begon M., Telford G., Woldehiwet Z., Birtles R.J. (2009). Delineating *Anaplasma phagocytophilum* ecotypes in coexisting, discrete enzootic cycles. Emerg. Infect. Dis..

[bib0030] Courtney J.W., Kostelnik L.M., Zeidner N.S., Massung R.F. (2004). Multiplex real-time PCR for detection of *Anaplasma phagocytophilum* and *Borrelia burgdorferi*. J. Clin. Microbiol..

[bib0035] de la Fuente J., Massung R.F., Wong S.J., Chu F.K., Lutz H., Meli M., von Loewenich F.D., Grzeszczuk A., Torina A., Caracappa S., Mangold A.J., Naranjo V., Stuen S., Kocan K.M. (2005). Sequence analysis of the msp4 gene of *Anaplasma phagocytophilum* strains. J. Clin. Microbiol..

[bib0040] Dumler J.S., Barbet A.F., Bekker C.P., Dasch G.A., Palmer G.H., Ray S.C., Rikihisa Y., Rurangirwa F.R. (2001). Reorganization of genera in the families Rickettsiaceae and Anaplasmataceae in the order Rickettsiales: unification of some species of *Ehrlichia* with *Anaplasma*, Cowdria with Ehrlichia and Ehrlichia with Neorickettsia, descriptions of six new species combinations and designation of *Ehrlichia equi* and ‘HGE agent’ as subjective synonyms of Ehrlichia phagocytophila. Int. J. Syst. Evol. Microbiol..

[bib0045] Dumler J.S., Choi K.S., Garcia-Garcia J.C., Barat N.S., Scorpio D.G., Garyu J.W., Grab D.J., Bakken J.S. (2005). Human granulocytic anaplasmosis and *Anaplasma phagocytophilum*. Emerg. Infect. Dis..

[bib0050] Dyachenko V., Pantchev N., Balzer H.J., Meyersen A., Straubinger R.K. (2012). First case of *Anaplasma platys* infection in a dog from Croatia. Parasit. Vectors.

[bib0055] Egenvall A.E., Hedhammar A.A., Bjoersdorff A.I. (1997). Clinical features and serology of 14 dogs affected by granulocytic ehrlichiosis in Sweden. Vet. Rec..

[bib0060] Engvall E.O., Pettersson B., Persson M., Artursson K., Johansson K.E. (1996). A 16S rRNA-based PCR assay for detection and identification of granulocytic *Ehrlichia* species in dogs, horses, and cattle. J. Clin. Microbiol..

[bib0065] Foley J.E., Nieto N.C., Massung R., Barbet A., Madigan J., Brown R.N. (2009). Distinct ecologically relevant strains of *Anaplasma phagocytophilum*. Emerg. Infect. Dis..

[bib0070] Franzen P., Aspan A., Egenvall A., Gunnarsson A., Karlstam E., Pringle J. (2009). Molecular evidence for persistence of *Anaplasma phagocytophilum* in the absence of clinical abnormalities in horses after recovery from acute experimental infection. J. Vet. Intern. Med..

[bib0075] Goodman J.L., Nelson C., Vitale B., Madigan J.E., Dumler J.S., Kurtti T.J., Munderloh U.G. (1996). Direct cultivation of the causative agent of human granulocytic ehrlichiosis. N. Engl. J. Med..

[bib0080] Kurtti T.J., Munderloh U.G., Andreadis T.G., Magnarelli L.A., Mather T.N. (1996). Tick cell culture isolation of an intracellular prokaryote from the tick *Ixodes scapularis*. J. Invertebr. Pathol..

[bib0085] Massung R.F., Levin M.L., Munderloh U.G., Silverman D.J., Lynch M.J., Gaywee J.K., Kurtti T.J. (2007). Isolation and propagation of the Ap-Variant 1 strain of *Anaplasma phagocytophilum* in a tick cell line. J. Clin. Microbiol..

[bib0090] Massung R.F., Levin M.L., Munderloh U.G., Silverman D.J., Lynch M.J., Kurtti T.J. (2006). Isolation of *Anaplasma phagocytophilum* strain Ap-variant 1 in a tick-derived cell line. Ann. N. Y. Acad. Sci..

[bib0095] Munderloh U.G., Blouin E.F., Kocan K.M., Ge N.L., Edwards W.L., Kurtti T.J. (1996). Establishment of the tick (Acari:Ixodidae)-borne cattle pathogen *Anaplasma marginale* (Rickettsiales:Anaplasmataceae) in tick cell culture. J. Med. Entomol..

[bib0100] Munderloh U.G., Jauron S.D., Fingerle V., Leitritz L., Hayes S.F., Hautman J.M., Nelson C.M., Huberty B.W., Kurtti T.J., Ahlstrand G.G., Greig B., Mellencamp M.A., Goodman J.L. (1999). Invasion and intracellular development of the human granulocytic ehrlichiosis agent in tick cell culture. J. Clin. Microbiol..

[bib0105] Munderloh U.G., Kurtti T.J. (1989). Formulation of medium for tick cell culture. Exp. Appl. Acarol..

[bib0110] Munderloh U.G., Madigan J.E., Dumler J.S., Goodman J.L., Hayes S.F., Barlough J.E., Nelson C.M., Kurtti T.J. (1996). Isolation of the equine granulocytic ehrlichiosis agent, *Ehrlichia equi*, in tick cell culture. J. Clin. Microbiol..

[bib0115] Munderloh U.G., Tate C.M., Lynch M.J., Howerth E.W., Kurtti T.J., Davidson W.R. (2003). Isolation of an *Anaplasma* sp. organism from white-tailed deer by tick cell culture. J. Clin. Microbiol..

[bib0120] Pedra J.H., Narasimhan S., Rendic D., DePonte K., Bell-Sakyi L., Wilson I.B., Fikrig E. (2010). Fucosylation enhances colonization of ticks by *Anaplasma phagocytophilum*. Cell Microbiol..

[bib0125] Pusterla N., Anderson R.J., House J.K., Pusterla J.B., Derock E., Madigan J.E. (2001). Susceptibility of cattle to infection with *Ehrlichia equi* and the agent of human granulocytic ehrlichiosis. J. Am. Vet. Med. Assoc..

[bib0130] Pusterla N., Pusterla J.B., Braun U., Lutz H. (1999). Experimental cross-infections with *Ehrlichia phagocytophila* and human granulocytic ehrlichia-like agent in cows and horses. Vet. Rec..

[bib0135] Rikihisa Y. (2011). Mechanisms of obligatory intracellular infection with *Anaplasma phagocytophilum*. Clin. Microbiol. Rev..

[bib0140] Scharf W., Schauer S., Freyburger F., Petrovec M., Schaarschmidt-Kiener D., Liebisch G., Runge M., Ganter M., Kehl A., Dumler J.S., Garcia-Perez A.L., Jensen J., Fingerle V., Meli M.L., Ensser A., Stuen S., von Loewenich F.D. (2011). Distinct host species correlate with *Anaplasma phagocytophilum* ankA gene clusters. J. Clin. Microbiol..

[bib0145] Scorpio D.G., Dumler J.S., Barat N.C., Cook J.A., Barat C.E., Stillman B.A., DeBisceglie K.C., Beall M.J., Chandrashekar R. (2011). Comparative strain analysis of *Anaplasma phagocytophilum* infection and clinical outcomes in a canine model of granulocytic anaplasmosis. Vector Borne Zoonotic Dis..

[bib0150] Silaghi C., Kauffmann M., Passos L.M., Pfister K., Zweygarth E. (2011). Isolation, propagation and preliminary characterisation of *Anaplasma phagocytophilum* from roe deer (*Capreolus capreolus*) in the tick cell line IDE8. Ticks Tick Borne Dis..

[bib0155] Simser J.A., Palmer A.T., Fingerle V., Wilske B., Kurtti T.J., Munderloh U.G. (2002). *Rickettsia monacensis* sp. nov., a spotted fever group rickettsia, from ticks (*Ixodes ricinus*) collected in a European city park. Appl. Environ. Microbiol..

[bib0160] Sunyakumthorn P., Bourchookarn A., Pornwiroon W., David C., Barker S.A., Macaluso K.R. (2008). Characterization and growth of polymorphic *Rickettsia felis* in a tick cell line. Appl. Environ. Microbiol..

[bib0165] Thomas R.J., Birtles R.J., Radford A.D., Woldehiwet Z. (2012). Recurrent bacteraemia in sheep infected persistently with *Anaplasma phagocytophilum*. J. Comp. Pathol..

[bib0170] Troese M.J., Carlyon J.A. (2009). *Anaplasma phagocytophilum* dense-cored organisms mediate cellular adherence through recognition of human P-selectin glycoprotein ligand 1. Infect. Immun..

[bib0175] Woldehiwet Z. (2006). Anaplasma phagocytophilum in ruminants in Europe. Ann. N. Y. Acad. Sci..

[bib0180] Woldehiwet Z. (2010). The natural history of *Anaplasma phagocytophilum*. Vet. Parasitol..

[bib0185] Woldehiwet Z., Horrocks B.K., Scaife H., Ross G., Munderloh U.G., Bown K., Edwards S.W., Hart C.A. (2002). Cultivation of an ovine strain of *Ehrlichia phagocytophila* in tick cell cultures. J. Comp. Pathol..

[bib0190] Zhou Z., Nie K., Tang C., Wang Z., Zhou R., Hu S., Zhang Z. (2010). Phylogenetic analysis of the genus *Anaplasma* in Southwestern China based on 16S rRNA sequence. Res. Vet. Sci..

[bib0195] Zweygarth E., Josemans A.I., Spickett A.M., Steyn H.C., Putterill J., Troskie P.C., Mtshali M.S., Bell-Sakyi L., Shkap V., Fish L., Kocan K.M., Blouin E.F. (2006). In vitro cultivation of a south African isolate of an *Anaplasma* sp. in tick cell cultures. Onderstepoort J. Vet. Res..

